# Commercial Smartphone-Based Devices and Smart Applications for Personalized Healthcare Monitoring and Management

**DOI:** 10.3390/diagnostics4030104

**Published:** 2014-08-18

**Authors:** Sandeep Kumar Vashist, E. Marion Schneider, John H.T. Luong

**Affiliations:** 1 HSG-IMIT—Institut für Mikro-und Informationstechnik, Georges-Koehler-Allee 103, 79100 Freiburg, Germany; 2 Laboratory for MicroElectroMechanical Systems (MEMS) Applications, Department of Microsystems Engineering (IMTEK), University of Freiburg, Georges-Koehler-Allee 103, 79110 Freiburg, Germany; 3 Sektion Experimentelle Anaesthesiologie, University Hospital Ulm, Albert Einstein Allee 23; 89081 Ulm, Germany; E-Mail: marion.schneider@uniklinik-ulm.de; 4 Innovative Chromatography Group, Irish Separation Science Cluster (ISSC), Department of Chemistry and Analytical, Biological Chemistry Research Facility (ABCRF), University College Cork, Cork, Ireland; E-Mail: j.luong@ucc.ie

**Keywords:** smartphone, devices, smart applications, personalized healthcare, mobile healthcare

## Abstract

Smartphone-based devices and applications (SBDAs) with cost effectiveness and remote sensing are the most promising and effective means of delivering mobile healthcare (mHealthcare). Several SBDAs have been commercialized for the personalized monitoring and/or management of basic physiological parameters, such as blood pressure, weight, body analysis, pulse rate, electrocardiograph, blood glucose, blood glucose saturation, sleeping and physical activity. With advances in Bluetooth technology, software, cloud computing and remote sensing, SBDAs provide real-time on-site analysis and telemedicine opportunities in remote areas. This scenario is of utmost importance for developing countries, where the number of smartphone users is about 70% of 6.8 billion cell phone subscribers worldwide with limited access to basic healthcare service. The technology platform facilitates patient-doctor communication and the patients to effectively manage and keep track of their medical conditions. Besides tremendous healthcare cost savings, SBDAs are very critical for the monitoring and effective management of emerging epidemics and food contamination outbreaks. The next decade will witness pioneering advances and increasing applications of SBDAs in this exponentially growing field of mHealthcare. This article provides a critical review of commercial SBDAs that are being widely used for personalized healthcare monitoring and management.

## 1. Introduction

Intensified research has focused on the development of advanced smartphone-based devices and applications (SBDAs) that offer cost-effectiveness and personalized mHealthcare, regardless of time and place. Current smartphones are increasingly loaded with sophisticated features and a variety of sensors, including light detectors, cameras, proximity sensors and fingerprinting. Thus, SBDAs are the most prospective and effective means of delivering mHealthcare to remote, resource-poor, private and public settings. In telemedicine, real-time diagnostics with spatio-temporal and pertinent information provided by smart devices can be kept on a secure central server and are accessible by healthcare professionals from remote locations. This feature is very critical for the monitoring and effective management of emerging epidemics and individual emergency cases [1].

Over 94% of the world population,* i.e.*, 6.8 billion people, are cell phone subscribers, and about 2.7 billion subscribers are Internet users [2]. Cell phone subscriptions might reach 8.5 billion by the end of 2016 [3] with 70% of smartphone users from developing countries. This enormous growth along with evolving technological features and cost reduction has vastly expanded the market size and new business opportunities. SBDAs will have a significant global impact in real-time on-site analysis and telemedicine opportunities in remote areas.

There are considerable growing applications of cell phone-based devices in bioanalytical sciences [4],* i.e.*, immunoassays [5,6,7,8,9], lateral flow assays (LFA) [10,11,12,13,14,15], electrochemical sensing [16,17,18,19], surface plasmon resonance-based biosensing [20], microscopy [21,22,23,24,25], flow cytometry [26,27] and colorimetric detection [28]. This manuscript aims to provide a comprehensive review of commercially available SBDAs (Table 1) and their future impact on personalized healthcare monitoring and management. It should be noted that only the major widely used SBDAs are described here; however, other devices and applications with similar key features are also commercially available. Most of the commercial SBDAs included in this article were well-known to us based on our ongoing research efforts in the field of personalized healthcare monitoring and management. We have intensive experience in the field of smartphone-based devices and have been developing a range of such devices for the colorimetric readout of* in vitro* diagnostics, which are under various stages of development. However, some of the mentioned SBDAs, such as those from GENTAG, Inc. (Washington, DC, USA), Mobile Assay, Inc. (Niwot, CO, USA) and Nonin Medical, Inc. (Plymouth, MN, USA), were searched from the respective websites of these companies that were googled on the Internet using the keywords of the companies’ names. The relevant information pertaining to the features of the mentioned SBDAs were taken from the information provided on the respective websites, the published literature and the product manuals. The prices of most of the SBDAs were taken directly from the websites, while in some cases, such as the SBDAs from Nonin Medical, Inc and Artificial Life, Inc. (Hong Kong, China), these were taken from the concerned persons in these companies.

**Table 1:** Commercial smartphone-based devices and smart applications (SBDAs) for personalized healthcare monitoring and management.

Company	Commercial SBDAs for Healthcare Monitoring and Management	Type of Mobile Platform	Clearance/Marking	Price (in US$)	Ref.
iHealth Lab, Inc. (Mountain View, CA, USA)	Wireless digital scale	iOS, Android	FDA, CE	69.95	[29]
	Wireless body analysis scale	iOS, Android	FDA, CE	109.95	-
	iHealth Lite wireless scale	iOS, Android	FDA, CE	79.95	-
	Wireless blood pressure (BP) monitor	iOS, Android	FDA, CE, ESH, EC Medical	99.95	-
	Wireless BP wrist monitor	iOS, Android	FDA, CE, ESH, EC Medical	79.95	-
	iHealth BP Dock	iOS, Android	FDA, CE, ESH, EC Medical	79.95	-
	iHealth MyVitals app	iOS, Android	-	Free	-
	iHealth Wireless Smart Gluco-Monitoring System	iOS, Android	FDA, CE, ISO 15197:2013	29.95	-
	iHealth Align	iOS, Android	FDA, CE	16.95	-
	iHealth Gluco-Smart app	iOS, Android	-	Free	-
	Wireless Pulse Oximeter	iOS, Android	FDA, CE	69.95	-
	iHealth SpO2 app	iOS, Android	-	Free	-
	iHealth Wireless Activity and Sleep Tracker	iOS, Android	-	59.95	-
AliveCor, Inc. (San Francisco, CA, USA)	AliveCor Heart Monitor	iOS, Android	FDA, CE	199	[30]
	AliveECG app	iOS, Android	-	Free	-
GENTAG, Inc.	Near-Field Communications (NFC) SensorLinkers	N.M. *	N.M. *	N.M. *	[31]
	NFC tags				
	NFC diagnostic skin patches				
	NFC sensors				
	NFC-radio frequency identification device (RFID) sensors				
	NFC immunoassays				
	NFC-Bluetooth weight management kit				
	Radar responsive (RR) tags				
	Cell phone-based home monitoring solutions				
	Transdermal glucose sensing and				
	monitoring system				
	Customizable spectroscopic radiation detection cell phone				
Mobile Assay, Inc.	Mobile diagnostic reader (mReader™)	iOS, Android, Windows	N.M. *	N.M. *	[32]
	Instantaneous Analysis™ software				
	Mobile Assay Cloud™ and Tracker™				
CellScope (San Francisco, CA, USA)	“CellScope Oto” digital Otoscope	iOS	FDA Class I device	N.M. *	[33]
	“CellScope Derm” Dermascope				
Holomic, LLC (Los Angeles, CA, USA)	Smartphone-based Holomic Integrated rapid diagnostic test reader (HRDR-200)	iOS, Android	FDA Class I device, ISO13485	N.M. *	[34]
	Holomic Google glass rapid diagnostic test reader	-	-		-
Runtastic GmbH (Pasching, Linz, Austria)	Runtastic Heart Rate Combo Monitor	iOS, Android, Windows	FCC, CE	94	[35]
	Runtastic GPS Watch and Heart Rate Monitor	iOS, Android, Windows	FCC, CE	202	-
	Runtastic LIBRA	iOS, Android, Windows	FCC, CE	175	-
Nonin	Onyx^®^ II 9560 Wireless Finger Pulse Oximeter	iOS, Android	FCC Class B digital device, ISO 10993-1, IEC 60601-1-2, SSP, HDP with security mode 2, IEEE11073, Continua	666	[36]
Artificial Life, Inc.	GluCoMo™	iOS, Android	-	0.99	[37]
	Opus-M™ Health: Neurodermatitis	iOS, Android	-	0.99	[38]
	Opus-M™ Health	iOS, Android	-	N.M. *	-

* N.M. not mentioned.

The personalized monitoring of health by the determination of weight, activity, sleep, heart rate, blood glucose diet and other parameters is immensely useful for the management of important chronic health conditions, such as diabetes, obesity, depression, ageing and mental health [1,39,40,41,42,43,44,45,46,47,48,49,50,51,52,53,54,55,56,57]. SBDAs will play an essential role in the rapidly evolving field of mHealthcare [58,59,60], as demonstrated by the connectivity of isolated remote healthcare laboratories [60,61], increased adherence to health monitoring, treatment regimen and medication [62,63,64,65,66,67], management of chronic diseases [68,69], general healthcare management by nurses [70], improved communication between healthcare professionals [71,72], providing education [73,74], screening of the community for a particular disease condition [75], prevention of infectious and sexually-transmitted diseases [76,77] and child obesity [78], spatiotemporal mapping of disease incidence [79] and improved adherence by parents to immunization schedules [80].

## 2. Commercial Smartphone-Based Devices and Smart Applications

### 2.1. iHealth Lab, Inc.

**Figure 1:**
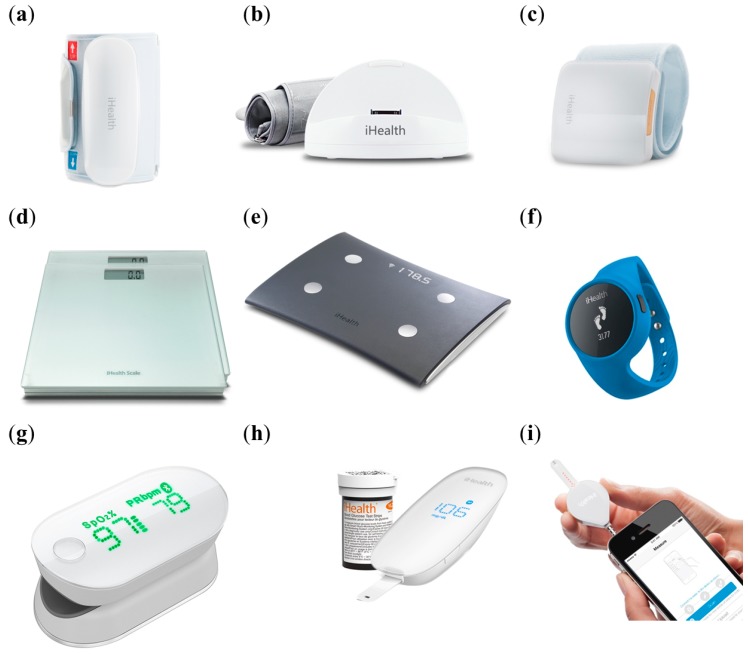
Smartphone-based devices (SBD) developed by iHealth Lab Inc. (**a**) Wireless blood pressure (BP) monitor; (**b**) iHealth BP dock; (**c**) wireless BP wrist monitor; (**d**) wireless digital scale; (**e**) wireless body analysis scale; (**f**) iHealth Wireless Activity and Sleep Tracker; (**g**) Wireless Pulse Oximeter; (**h**) iHealth Wireless Smart Gluco-Monitoring System; (**i**) iHealth Align. Reproduced with permission from iHealth Lab Inc.

iHealth Lab Inc. [29] provides several prospective wireless smartphone-based devices (SBDs) (Figure 1), such as a body analysis scale, digital scale, blood pressure (BP) wrist monitor and BP monitor, besides an integrated mobile application (iHealth MyVitals app). The wireless scale measures, stores, monitors and shares the weight and body mass index (BMI) using Bluetooth^®^. Cleared by the Food and Drug Administration (FDA) and Conformité Européenne (CE), the newer version is the wireless body analysis scale, consisting of four electronic sensors and a set of proprietary algorithms, to measure body compositions. This stand-alone device can simultaneously analyse the weight, body fat, lean mass, muscle mass, bone mass, body water, daily calorie intake, BMI and visceral fat rating of individuals. Both scales can be used by multiple users on all of the iOS and Android devices. The wireless body analysis scale and the wireless scale are sold for US$ 109.95 and US$ 69.95, respectively. The wireless BP monitor measures and tracks the systolic/diastolic values, heart rate and pulse wave. The results presented as visual charts are generated based on historical averages and World Health Organization (WHO) classifications. A BP dock is used for transforming an iOS device into a personalized BP monitor. The wireless BP monitor, with FDA clearance, ESH and EC Medical-certified CBD, can be attached to a small cuff on the wrist to serve as a motion sensor with high precision. The price of the wireless BP monitor, the wireless BP wrist monitor and the BP dock is US$ 99.95, US$ 79.95 and US$ 79.95, respectively. The iHealth MyVitals app monitors and shares the data pertaining to weight, blood pressure (BP), diet, physical activities and sleep. As an aid in personalized health management, it enables the users to set goals, view health trends, track progress, log food intake and activities and share the personalized information with families, friends and/or doctors. Additionally, it enables the creation of plans and the setting of reminders.

Another recent product is the Wireless Smart Gluco-Monitoring System, cleared by the United Stated Food and Drug Administration (FDA) and Conformité Européenne (CE), which meets the ISO 15197:2003 requirements for* in vitro* blood glucose monitoring. Priced at US$ 29.95, the meter can be used with the iHealth Gluco-Smart App, which empowers the users to effectively manage their diabetes rather easily and conveniently. The user is simply asked to create a unique iHealth ID, enabling access to free and secure cloud services. The app allows the user to take and log glucose measurements anywhere to view the trends and statistics for up to 90 days. The glucose meter, powered by a built-in, rechargeable battery, is sufficient for up to 200 tests. This sleek meter with an easy-to-read light emitting diode (LED) display performs glucose measurements within the diabetic pathophysiological range of 1.1–33.3 mol/L in 5 s using 0.7 μL of fresh capillary whole blood. The meter is connected via Bluetooth to the smart app on iOS or Android devices equipped with personalized healthcare tools for tracking and effective management of blood glucose. The app automatically determines the remaining quantity of test strips in the vial and expiration information and alerts the user for a new vial. The patient saves up to 500 blood glucose test results on the meter offline, uploads the results to the iOS device app via the “upload” button to track the trends and to set up medication alerts and insulin reminders. It also alerts when the test strip expires, thereby preventing the potential error of false evaluation. The results can be shared with family members, friends and/or doctors. The most recently commercialized glucometer by iHealth is iHealth Align, which is FDA-cleared, CE-marked and can be purchased off the shelf for just US$ 16.95. This smallest glucometer plugs directly into the headphone jack of the smartphone and displays results instantly on the screen. It employs the same iHealth Gluco-Smart app and analytical features of the Wireless Smart Gluco-Monitoring System.

The Wireless Pulse Oximeter, sold at US$ 69.95, FDA-cleared and CE-marked, determines the blood oxygen saturation (SpO2) and the pulse rate (BPM) using a lightweight and portable device to perform non-invasive and rapid measurements at the fingertip. The technology involves the shining of two light beams into the small blood vessels or capillaries of a finger, to determine the amount of blood oxygen. It displays the measurement on the oximeter’s screen and records the result in the device’s memory. With wirelessly connection via Bluetooth^®^ to the iHealth SpO2 app on iOS and Android devices, the user can view the trends and organize and share such information. SpO2 reflects the amount of oxygen carried out by the blood as a percentage of the maximum carrying amount. The normal SpO2 is 96%–99% for a healthy individual, but is affected by high altitudes and other factors. Similarly, the normal resting pulse rate of 60–100 bpm is also affected by physical activity, fitness level, body weight, emotional state, medication and body position. The SpO2 measuring range is 70%–99%, while the average root mean square accuracies for SpO2 ranges of 80%–99% and 70%–79% are ±2% and ±3%, respectively. Similarly, the pulse rate measuring range is 30–250 bpm, while the pulse rate accuracies for the pulse rate ranges of 30–99 bpm and 100–250 bpm are ±2 bpm and ±2%, respectively. The device is a very useful tool for athletes and patients with breathing difficulties (pulmonary dysfunction), COPD (chronic obstructive pulmonary disease), coronary heart diseases and other vascular health conditions.

The iHealth Wireless Activity and Sleep Tracker, available for US$ 59.95, is a watch-shaped device that tracks the personalized daily activity and sleep. It is connected to the iHealth MyVitals app on iOS and Android devices via Bluetooth 4.0 with a low energy requirement. It is sweat, rain and splash proof and worn like a wrist watch or attached to the waist with a clip. It consists of a three-axis accelerometer to detect the 3D-motion patterns and provides information related to the steps taken by the users, burnt calories, travelled distance, sleeping hours and sleeping efficiency. The iHealth Tracker is powered by a rechargeable built-in battery that typically lasts over five days. With an accuracy of 95%–97%, the device is made of hypoallergenic and skin-friendly TPU rubber,* i.e.*, latex- and PVC-free. The smart app enables the setting of goals and silent vibrating alarms apart from viewing the trends, organizing records and information sharing within a social network. As a true personalized healthcare tool, it enables the users to adapt their lifestyle by keeping track of the defined general health parameters with physical activities, diet and other measures.

### 2.2. AliveCor, Inc.

**Figure 2:**
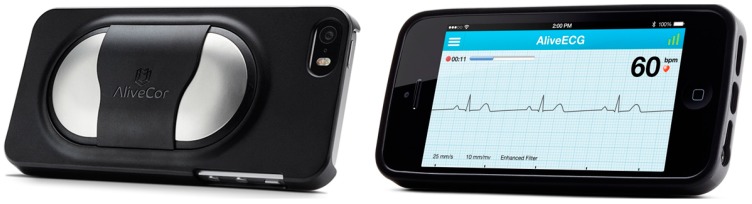
AliveCor Heart Monitor. Images provided by Rebecca Phillips, AliveCor Inc.

AliveCor, Inc. [30] was founded by Dr. David Albert, who came out with the idea of iPhone-based electrocardiogram (ECG). The FDA-cleared and CE-marked device (Figure 2) is cleared for sale in the U.S., the U.K. and Ireland. It allows medical professionals to record, display, store and evaluate single-channel ECG rhythms and heart rates. The users can record their personalized ECG with immediate interpretation. The ECG analysis provides an expert review of ECGs by U.S. board-certified cardiologists, U.S.-based cardiac technicians and U.K.-based cardiac physiologists. The device sold at US$ 199 is a Class II medical device that snaps onto an iPhone 5/5s or Samsung S4 like a case and wirelessly communicates with the SBDA,* i.e.*, AliveECG is running on the same smartphone unit. The AliveECG app is free to download for iPhone and Samsung users, but requires an initial account set-up. Personalized and encrypted ECG data and information are stored via cloud computing on a secure server, which can be remotely accessed as desired. The device runs on a 3-V coin cell battery that takes up to 10,000 ECGs of a 30-s duration. The Lead I ECG is generated by resting the electrodes on the fingers from each hand or on the chest. The app senses the skin contact with the sensors and initiates the ECG recording after establishing an acceptable connection. The AliveCor Heart Monitor employs a proprietary technology to convert electrical impulses to ultrasound signals, which are transmitted to the smartphone’s microphone. The enhanced filtering technology minimizes artefacts, yielding a high fidelity tracing comparable to the Lead I on standard ECG machines. This relatively cost-effective device can be used to evaluate the patient’s heart rate and rhythm. Its clinical accuracy has been demonstrated in several clinical trials; perhaps this is an educational tool for a subject with heart conditions to obtain rapid and accurate screening and recording of ECGs. The availability of a detailed arrhythmia library (with real-life sample ECGs), diagrams of cardiac anatomy and a breakdown of the ECG waveform provides enriched heart information that motivates the users to take care of their heart health. The users bear no extra cost, since the device is paid for by Medicare and other private insurance companies in the U.S.

### 2.3. GENTAG, Inc.

**Figure 3:**
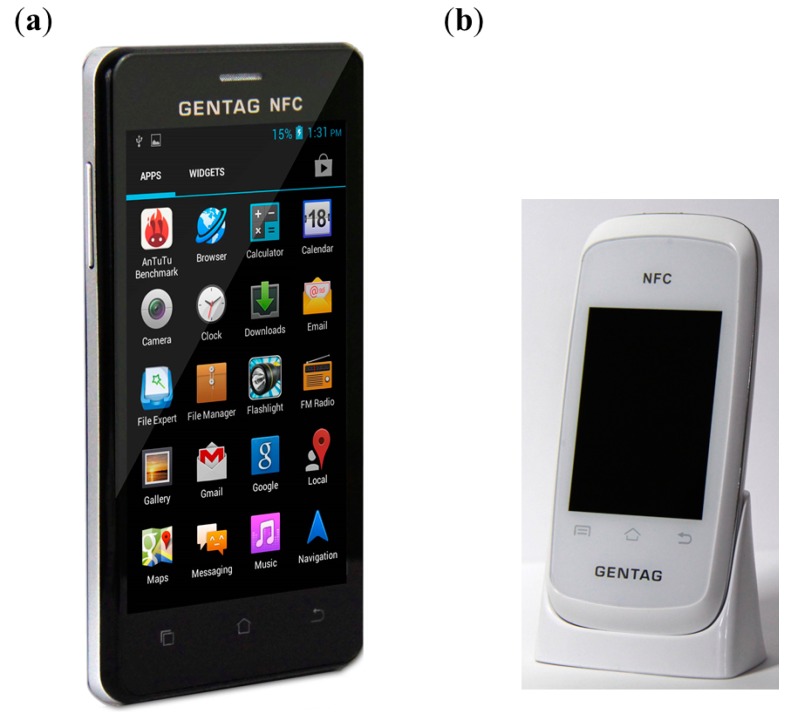
(**A**) NFC smart cell phone and (**B**) NFC SensorLinker by GENTAG, Inc. Images provided by Dr. John P. Peeters, GENTAG, Inc.

GENTAG [31], a leading company in mHealthcare, offers near-field communication (NFC)-based, low-cost, disposable wireless sensors. The device can be read and spatio-temporally tagged by NFC-capable smartphones (Figure 3A), tablets or personal computers for healthcare, diagnostics and mobile-to-mobile (M2M) applications. GENTAG’s proprietary technology provides a lower cost alternative compared to Bluetooth^®^, enabling the reading of NFC-based sensors or other customized sensors from a distance of one inch to 15 miles. Wireless NFC SensorLinkers are portable, lightweight (74 g), battery-powered devices for home medical monitoring applications, smart homes and machine-to-machine applications (Figure 3B). The technology can include any certifications, including ISO13485, MDDS Class I FDA clearance and Continua certification. It employs the NFC NXP PN544 Reader integrated circuit (IC) equipped with a proprietary long-range antenna. It consists of 2.4 GHz Wi-Fi 2–4 GHz with a long-range antenna, Bluetooth 4 Dual Mode, a USB port for data transfer, a lithium ion rechargeable battery, a rechargeable cradle and a USB charger. The SensorLinker runs on any SIM-based GPRS or 3G WCDMA cellular network and can be paired with existing Bluetooth devices or bundled with custom-made NFC sensors. The technology is used for wireless elderly care/child/compliance/medication monitoring, wireless hospital discharge kits and hybrid Wi-Fi/Bluetooth/NFC sensor applications. It can be easily integrated with FDA-cleared Bluetooth devices for measuring BP, weight, diabetes, COPD and other medical conditions. Implantable NFC sensors and devices, such as pacemakers, with an ultra-low power, can also be read and monitored directly via this technology.

The company utilizes NFC to develop disposable wireless skin patches, personal drug delivery systems and smartphone-based sensors (for asthma prevention, chemical, biological or radiation threat detection). The wireless skin patches can be waterproof, showerproof and non-allergic. They have a unique ID and employ a low-cost passive technology (without any battery), an FDA-cleared adhesive lasting 1–2 weeks and a 13.56 MHz frequency, which is the most widely used global standard in healthcare. Employing a radar-responsive (RR) tag for precise wireless geo-tagging over several miles, “wide area non-GPS wireless geolocation and geofencing” technology may lead to several prospective applications. The RR tags are used with smart diagnostic skin patches to monitor and locate patients with special needs, such as Alzheimer’s dementia. The GENTAG capabilities include an NFC-radio frequency identification device (RFID) sensor, an NFC tag for preventing counterfeit drugs or products, NFC diagnostic skin patches for fever monitoring, drug delivery, glucose monitoring, post orthopaedic surgery/post-hospital discharge monitoring and the prevention of hospital errors (due to mismatches from patient-surgery, patient-drug delivery/medication, mother-baby,* etc.*), NFC sensors for temperature, radiation, chemicals and pressure, an NFC immunoassay for prostate cancer, smartphone-based home monitoring solutions, e.g., an NFC thermometer, NFC custom blister packs and medication sensors, remote wireless monitoring of elderly or at-risk patients directly in their homes by taking BP, fever and medication data, an NFC-Bluetooth^®^ weight management kit and RR tags.

GENTAG, Inc., and MacroArray Technologies, LLC (Villanova, PA, USA) jointly developed a smartphone-based disposable immunoassay procedure for urine samples to diagnose prostate cancer using the proprietary PCADM-1 biomarker of MacroArray Technologies, LLC. It is a non-invasive and highly cost-effective consumer-based test for the frequent testing for prostate cancer. Since more frequent measurements are possible, this procedure might potentially replace the individual and isolated determination of the prostate-specific antigen (PSA)-based blood test during hospital screenings. Due to the influence of other physiological factors in a patient, the reliability of the PSA-based blood tests is questionable [81].

GENTAG, Inc., has obtained a patent for a smartphone-based painless transdermal glucose sensing and monitoring system using disposable skin patches with wireless sensors [19]. Such patches could be more cost-effective than several existing glucose monitoring devices [82,83,84] and offer an annual savings of US$ 3,000 and US$ 300 for type 1 and type 2 diabetics per patient. In particular, it obviates finger-pricking technology. Apart from the glucose measurement, the smartphone can be programmed for insulin delivery and the geolocation of patients in an emergency.

GENTAG, Inc., has also demonstrated a customizable spectroscopic radiation detection cell phone, based on the sensor technology from eV Products, Inc. (now i3 Electronics, Endicott, NY, USA), to discriminate hazardous γ-rays from normal γ-rays. The combination of this technology with GENTAG’s wireless sensing technologies,* i.e.*, NFC reader and RR tags, offers software-based remote geolocation and monitoring.

### 2.4. Mobile Assay, Inc.

**Figure 4:**
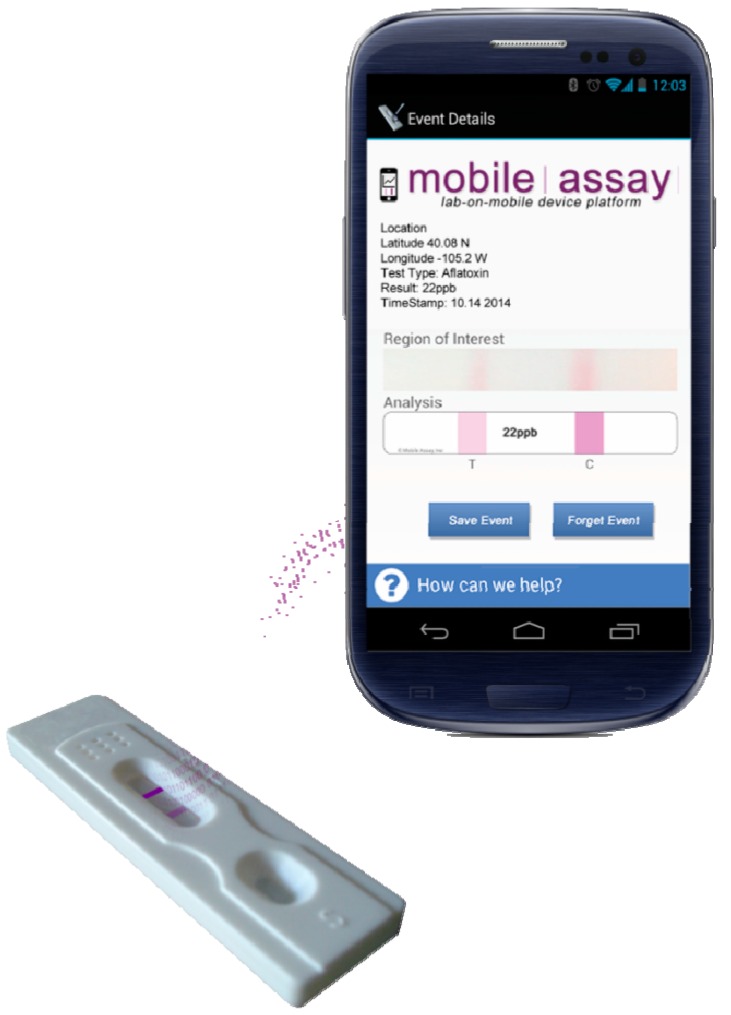
The mobile diagnostic reader (mReader™) by Mobile Assay, Inc. Image provided by Michael Williams, Mobile Assay, Inc.

Of interest is a cost-effective mobile diagnostic reader (mReader™) from Mobile Assay, Inc. [32] (Figure 4), based on the principle of mobile image radiometry (MIR) in combination with Instantaneous Analysis™ software. The reader provides fast and accurate tracking and quantification of inexpensive rapid lateral flow test strips by a smartphone or tablet [12,13]. Without any additional attachments, the reader can read multiple tests simultaneously with accurate detection down to 1 ppb, significantly more sensitive than the human eye. Based on a built-in advanced light level compensation and camera linearity, the reading is unaffected by ambient lighting fluctuation. The geo-tagged and time stamped results are then uploaded to the mobile diagnostic cloud via Wi-Fi or a cellular network with the push of a button for data analysis and storage. The company provides secure “sign in” to its portal with secure encryption and data storage techniques. The results stored in the cloud are analysed for trend analysis, outliers and reports specific to a test type or area where the test is performed. The results can be thumb tacked to a map, and both internal and external compliance reports can be generated in any desired formats. The quantification is achieved instantaneously through the image analysis of the dye signal on the test strips, where the MIR subtracts the background noise, selects the signal bands and provides the pixel density ratio. The sample is dispensed at the specified area on the test strip and taken by capillary action together with the dye-conjugated antibodies, specific for the target analyte, across the test and control areas. There are two bands for the sample without the analyte, due to the dye binding at the test and the control areas. With the analyte, the relative intensity of the test band as determined by MIR increases with increasing analyte concentrations. The Data Collection Module transfers real-time data to the Mobile Assay Cloud™, which can be accessed locally via Tracker™ or remotely via Mobile Assay Cloud™. The Tracker Manager™ can also send text alerts to the clients. The smart application runs on Apple, Android and Windows platforms. The developed technology has been demonstrated for detecting drugs (e.g., cocaine and benzoylecgonine), food pathogens, water content and aflatoxin. It is capable of detecting 0.1–300 ng/mL of cocaine and 0.003–0.1 ng/mL of benzoylecgonine apart from food pathogens [15], to allow the rapid and efficient tracking of the origin and severity of outbreaks. The technology is a boon to food producers for the prevention of a wide-scale distribution of contaminated food by taking appropriate measures at an early stage. This will lead to tremendous cost-savings considering about 48 million cases of foodborne illness annually in the United States [85] with an annual financial burden of US$ 51–78 billion [86]. It also leads to increased agricultural productivity by significantly reducing crop losses based on the early detection and tracking of *Botrytis cinerea*, a fungus that causes significant damage to plants and flowers.

### 2.5. CellScope

**Figure 5:**
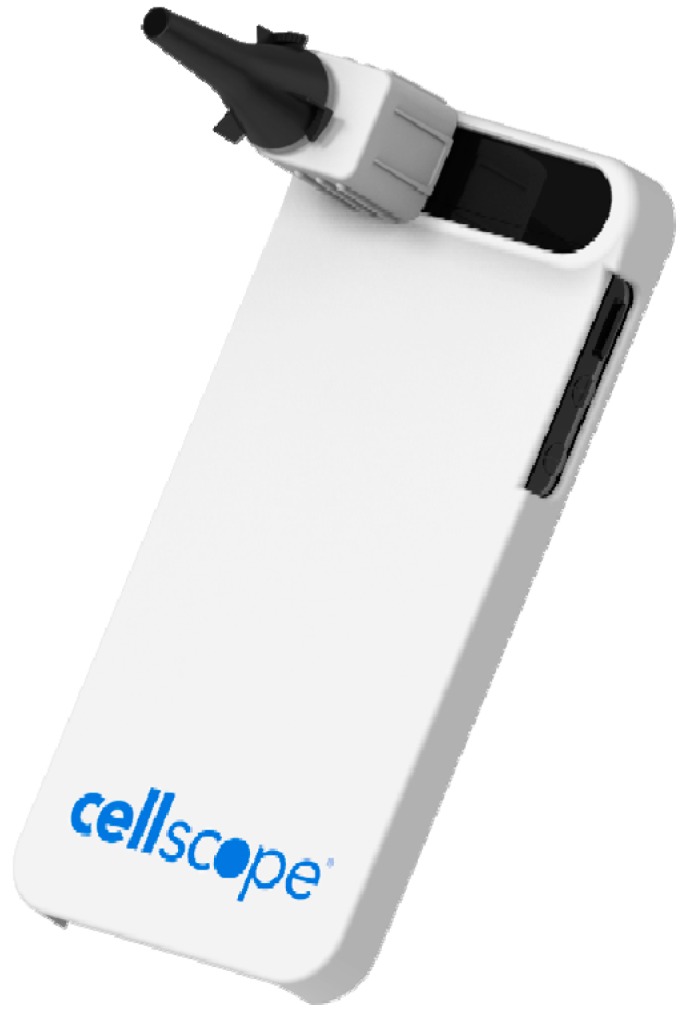
CellScope Oto by CellScope. Image provided by Cori Allen, CellScope, Inc.

CellScope [33], a startup company in San Francisco, is founded in Prof. Daniel Fletcher’s research lab at the University of California, Berkeley, by Erik Douglas and Amy Sheng. The company joined Rock Health’s inaugural class of healthcare startups and secured a seed investment from Khosla Ventures. The business focuses on easy-to-use personalized healthcare tools for home diagnostic applications. CellScope has developed two optical attachments to modify a smartphone into a diagnostic-quality imaging system for healthcare and consumer skincare. As a clip-on device or a digital Otoscope, “CellScope Oto” takes visual images of the middle ear to probe “ear infection” (Figure 5). It comprises a specifically designed iPhone case, scope attachment, scope case, five tip cases and an iPhone app along with a HIPAA-compliant website for reviewing, comparing and transmitting the results of the ear examination. The device and the smart app help parents to minimize doctor visits. High quality images of the ear canal and eardrums uploaded on the CellScope’s web platform can be remotely accessed by a doctor for diagnosis, treatment and monitoring. The device is useful for school or day care facilities to screen sick children for ear infection. It is highly cost-effective, taking into account 30 million doctor visits annually in the United States. Similarly, the “CellScope Derm” is another clip-on Dermascope for remote diagnosis of patient’s skin conditions based on the capture and transmission of highly-magnified diagnostic quality images. It is equipped with an illumination system and lower-magnification optics to capture a wider field. Both of the devices are in pilot testing by doctors around the San Francisco Bay Area.

### 2.6. Holomic LLC

**Figure 6:**
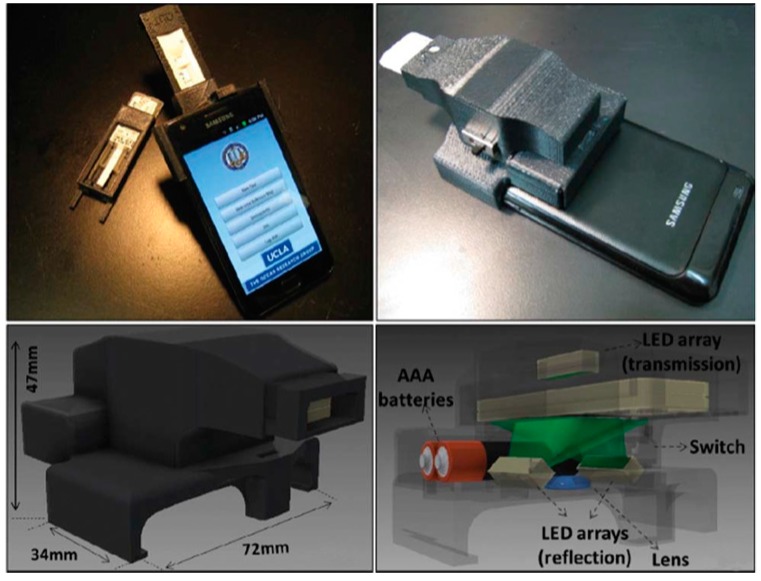
Smartphone-based Holomic Integrated rapid diagnostic test reader (HRDR-200) developed by Holomic, LLC [11]. Reproduced with permission from the Royal Society of Chemistry.

Holomic LLC [34] was formed in 2011 to commercialize the SBDs developed at Prof. Ozcan’s laboratory at UCLA, USA. The main product is a light-weight, handheld, economical, quantitative and universal smartphone-based Holomic Rapid Diagnostic Test Reader (HRDR-200) to perform lateral flow immunoassay tests (even in different sizes and formats) for POC, telemedicine and public health monitoring. The device is compliant with ISO13485 and registered with the FDA as a Class I medical device. It is available for sale globally, but can only be used for research in the U.S. The HRDR-200 comes with an integrated reader housing, a smartphone, a software application and access to Holomic Cloud Services and Holomic Test Developer. The reader also has access to electronic health records (EHRs) for real-time data collection via a secure Holomic cloud service. The cloud services are HIPAA compliant and compatible with HL7 standards. They are provided on a subscription basis by Holomic’s partner, Soft Computer Corporation. The HRDR-200 can read lateral flow, flow-through and dipstick tests with a high accuracy to provide quantitative or qualitative analysis along with the barcode/QR test and patient data entry, as well as test and lot number identification. The device has chromatographic and fluorescent options for the readout and can be connected to laptops, PCs, printers and EHRs via wireless, Bluetooth and USB, as demonstrated recently by Prof. Ozcan’s group [11]. The demonstrated rapid diagnostic test (RDT) reader includes an inexpensive plano-convex lens, three LED arrays (two located underneath the RDT tray for reflection imaging and one at the top for transmission imaging) and a low-cost microcontroller. It can be powered by external batteries or the smartphone battery via a USB connection, and it quantitatively analyses various types of lateral flow immunoassays or RDTs in reflection or transmission imaging modes under diffused LED illumination (Figure 6). The raw images are digitized within <0.2 s via a smart application, and the resulting information is stored locally and the test results shared as an evaluation report with a central server. The RDT reader provides a precise and reproducible evaluation of RDTs with very high sensitivity as the minor colour signal variation, which cannot be observed otherwise by the naked eye. The RDT reader’s software can be installed on Android- and iOS-based smartphones. The test results are shared with the central database and accessed through the same application or a remote computer using web browsers. The software application provides a dynamic spatio-temporal map and real-time statistics for various diseases (e.g., malaria, human immunodeficiency virus and tuberculosis) and conditions that can be diagnosed by RDTs, thereby providing the desired information to healthcare professionals and policy makers to monitor, track and analyse emerging diseases and outbreaks. Holomic also provides custom readers with specific requirements from the end-users. It also provides the test developer PC software application to the developers and manufacturers of rapid diagnostic tests for an in-depth insight into test data and reader calibration.

### 2.7. Runtastic GmbH

**Figure 7:**
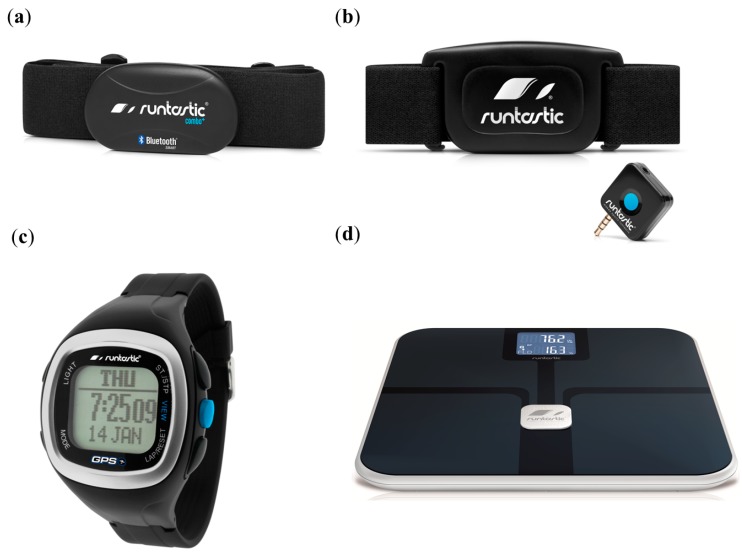
SBD developed by Runtastic GmbH. (**a**) The Bluetooth low-energy chest strap of the Runtastic Heart Rate Combo Monitor; (**b**) chest strap and dongle of the Runtastic Heart Rate Monitor; (**c**) Runtastic GPS Watch; (**d**) Runtastic LIBRA weighing scale. Images provided by Johannes Knoll, Runtastic GmbH.

Runtastic GmbH [35] has developed various FDA-cleared and CE-marked SBDAs for sports and fitness (Figure 7), such as the Runtastic Heart Rate Combo Monitor, Runtastic LIBRA weighting scale, Runtastic GPS Watch and Heart Rate Monitor, as well as various accessories. The Runtastic Heart Rate Combo Monitor, available for US$ 94, is equipped with a transmitter and a chest strap, which are powered by lithium batteries and are splash-proof. However, it must be protected from a very strong magnetic field, which causes interference, and the device is not intended for people with limited physical, sensory or mental abilities and/or who lack experience/knowledge. Users with pacemakers should only use the device after consulting their doctor. The device is designed for heart rate measurement as a training tool instead of a medical device. It employs a low-power Bluetooth^®^ smart technology for the transmission of the heart rate from the chest strap (transmitter) to the smartphone. The chest strap also sends the signal at a transmission frequency of 5.3 kHz, compatible with most training devices in the gym, provided a separate Runtastic receiver (charged by lithium battery) is used. The transmitter, preferably with its contacts moistened by an ECG gel, is connected to the strap by two push buttons. The elastic strap is then adjusted so that the sensors contact the chest below the pectoral muscles. The Runtastic app needs to be downloaded on the smartphone in addition to the setting up of the personalized account before the monitor’s results appear on the smartphone. Once the Bluetooth connection is established between the monitor and the smartphone, the device displays heart rate real-time results during exercise via the Runtastic app. An alternative is to plug the Runtastic receiver into the headphone jack of the smartphone, which activates the receiver immediately to provide real-time results from the transmitter after enabling the receiver (ON) in the smartphone settings. The receiver battery lasts about 2.5 years with a daily operation of 1 h. The transmission can switch between coded and uncoded transmission modes by button pressing for 5 s. The encoded transmission is less susceptible to interference from other similar transmitters in operation. The uncoded transmission is ideal, as it employs a frequency used by most fitness equipment, such as treadmills and cross trainers. The chest strap might contain latex components that trigger allergic reactions, such as skin irritations and redness, and its use must be discontinued immediately in this situation.

The Runtastic GPS Watch and the Heart Rate Monitor, sold at US$ 202, track pace, speed, duration, laps, calories and heart rate together with elevation change and target heart rate training zones. The watch has a battery life of up to 14 h, a reliable compass with navigation functions, a customizable display, a night light and other functions. Both the watch and the monitor are just 57.5 g and 60 g, respectively. The device, charged via a USB cable, transfers all of the fitness statistics and graphs to the user’s account on the Runtastic website [35] to a computer with an active Internet network. The website provides a detailed analysis of the results, which can be shared online with the Runtastic community.

The Runtastic LIBRA, sold at US$ 175, is a digital body analysis scale that measures the overall weight (up to 180 kg), body fat and water percentage, muscle and bone mass, BMI, basal metabolic rate (BMR) and active metabolic rate (AMR) (calories burnt). It is based on the principle of bioelectrical impedance analysis (BIA) for immediate measurements of body metrics using an imperceptible, completely harmless and safe alternating current. Muscle tissues and water have good electrical conductivity,* i.e.*, low resistance, while bone and fat tissues have low conductivity or high resistance. The measurements are transferred from the scale to the smartphone or the tablet by energy-efficient Bluetooth^®^ smart technology from a distance as far as 25 m via the Runtastic Libra app, freely available from the iTunes store. The scale accommodates up to eight users and automatically assesses their personal body metrics. It is composed of a highly resistant glass plate coated with indium tin oxide (ITO) electrodes, weighs 2.5 kg and employs 3 × 1.5 V AAA alkaline batteries. The users can log on to their account at the Runtastic website [35], to generate detailed statistics and an in-depth analysis using their body metrics. However, the scale should not be used by persons with medical implants (such as pacemakers), pregnant women and children below 10 years old. Areas with very strong magnetic fields should be avoided, as they interfere with the signal transmission. The company also provides the Runtastic USB Power Bank with a 5600-mAh capacity, a rechargeable integrated LED flashlight via a mini-USB cable, with a nominal cost of US$ 40. It can be used for charging various Runtastic devices for extended session tracking anytime and anywhere.

### 2.8. Nonic Medical, Inc.

**Figure 8:**
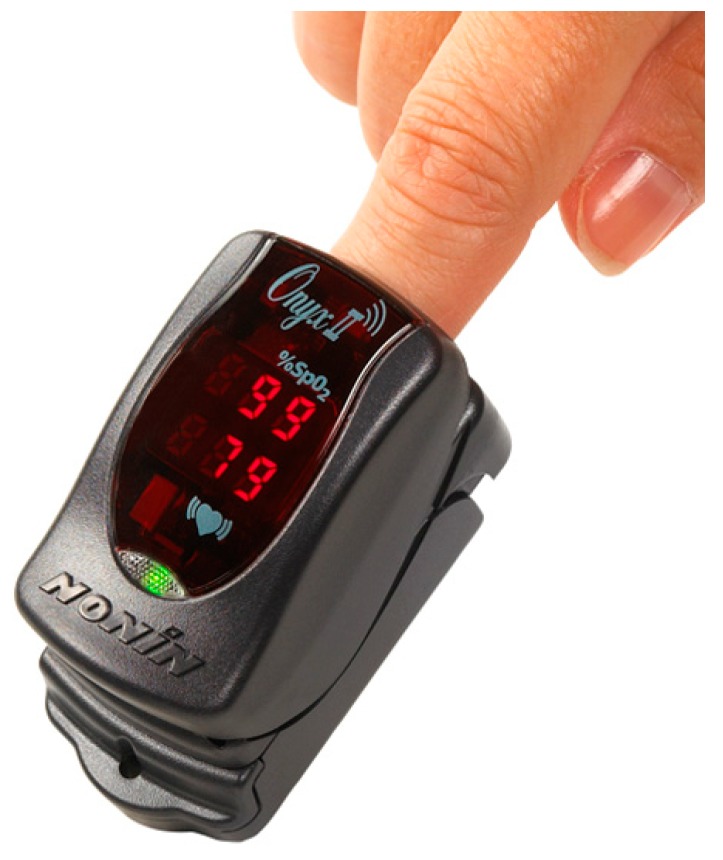
The Onyx^®^ II Model 9560 Finger Pulse Oximeter developed by Nonin Medical, Inc.

Nonin Medical, Inc. [36], has more than two decades of experience in the manufacturing of pulse oximeters with the desired clinical accuracy [87,88,89]. The company has developed an Onyx^®^ II Model 9560 Finger Pulse Oximeter (Figure 8), which is a lightweight (63 g) and compact wireless device for the measurement of SpO_2_ and the pulse rate of well or poorly perfused patients, such as those with COPD, congestive heart failure and asthma. It can be purchased at a price of US$ 666. The technology is based on the non-invasive pulse oximetry method [90,91] that involves the passing of red (660 nm) and infrared (910 nm) light through perfused tissue to detect the fluctuating signal due to arterial pulses. The device measures the SpO_2_ levels from the difference in colour by determining the absorbed red and infrared light ratio. The well- and poorly-oxygenated blood, respectively, are characterized by bright red or dark red. It is extended for personalized use apart from its routine use in hospitals, clinics, skilled nursing facilities and long-term care facilities. The device can perform the measurements on a diverse range of subjects with fingers with a thicknesses of 0.8–2.5 cm. It can be used in paediatrics and adults having light to dark skin tones and good to low perfusion. It can take the readings wirelessly up to 100 m and can store a minimum of 20 single-point measurements. Moreover, it involves a sophisticated SmartPoint algorithm for the automated determination of SpO_2_ and the pulse rate apart from employing a new power-saving feature based on the automatic adjustment of the transmitted power depending on the distance between the device and the main unit. In addition, the proprietary PureSAT signal processing technology used in the device enables precise measurements by removing the noise, artefacts and/or interferences. The device runs on two AAA batteries that are sufficient for over one year of operation. The SpO_2_ and the pulse rate measuring ranges are 0%–100% and 18–321 BPM, respectively. The average root mean square accuracies for a SpO_2_ range of 70%–100% and a pulse rate of 20–250 BPM are ±2 and ±3, respectively.

The Bluetooth radio in Onyx^®^ II Model 9560 complies with version 2.0 of the Bluetooth Specification, which supports the Serial Port Protocol (SPP), the Health Device Profile (HDP) with security Mode 2 (service level enforced), IEEE11073 and Continua. It is certified to Microsoft^®^ HealthVault^®^, a free online platform that communicates and receives data from the device for personalized healthcare management. The device complies with the IEC 60601-1-2 for electromagnetic compatibility, Part 15 of the Federal Communications Commission (FCC) Rules as a Class B digital device, and ISO 10993-1. However, Federal Law (USA) restricts this device to sale by or on the order of a licensed practitioner.

### 2.9. Artificial Life, Inc.

**Figure 9:**
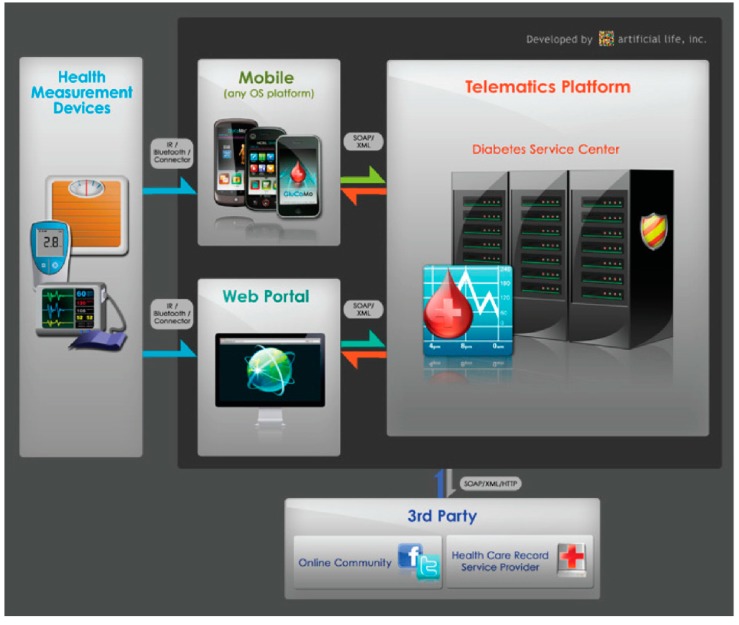
GluCoMo™ developed by Artificial Life, Inc. Reproduced with permission from Ada Fong, Artificial Life, Inc.

Artificial Life, Inc. [38], has developed a highly prospective SBDA, called GluCoMo™ [37] (Figure 9), an electronic diary and a reminder system for diabetics targeted towards diabetic monitoring and patient coaching. It can be downloaded from the iTunes store for only US$ 0.99. Its telematics feature is compatible with various platforms, such as iOS, Android, Symbian, Blackberry, Windows Phone 7 and Java. The SBDA allows diabetic patients to monitor their blood glucose levels, diet, insulin intake and other activities. It consists of customized mobile client applications, an interactive web portal and a secure telematics platform. Pertinent data and information are circulated among patients, doctors, hospitals and other healthcare providers. The data in the client application are kept updated on a central database through, e.g., 3G, 4G or Wi-Fi networks, which are accessible by authorized healthcare professionals. This feature allows scheduled and real-time communication between doctors and patients and also inter-hospital communication, where doctors can share advice, medical opinions and instantaneous diagnostics. It also sends warnings to users if dangerous trends are detected and, additionally, reminds them to keep track of their activities. The application uses various methods for encryption, including secure authentication, firewall, virus protection, secure transport and data storage. It enables diabetics to engage in effective lifestyle intervention by adjusting or scheduling activities after reviewing their entry history in a simple, but informative, graph. Users can set up automatic reminders to alert them of various health activities and engage in online interaction and sharing via built-in forums. Additionally, the users have access to an informative handbook to acquire diabetic facts.

The company has also launched Opus-M™ Health: Neurodermatitis, which can be downloaded from the iTunes store for only US$ 0.99. It is an electronic diary and monitoring tool for patients and caregivers of those suffering from lichen simplex chronicus or neurodermatitis, a serious skin disorder. The users can create the entries of photos taken by the iOS device’s camera to keep track of the neurodermatitis-related information, such as itching, pain, redness and weep and visualize their condition and the healing process over time using the slideshow function. The software includes simple and easy-to-use data input for photo and text entries to provide an interactive graph for tracking and visualization. The PDF data summary can be transferred to a computer via iTunes sharing and forwarded by emails to responsible doctors and caregivers. The users also get the desired skin care tips that minimize the effect of neurodermatitis. Another product is Opus-M™ Health, a family of healthcare apps that meets various medical needs of the public, so that the patients and their caregivers can effectively manage and keep track of their medical conditions. It greatly facilitates communication between the patients, doctors, hospital and caregivers. The company is developing Opus-M™ HIV/AIDS, an application under Opus-M™ Health, dedicated to care management for HIV/AIDS patients and their community.

## 3. Conclusions

SBDAs have demonstrated the successful convergence of various scientific disciplines, such as engineering, biomedical sciences, chemistry and physical sciences,* etc.*, to develop innovative technologies for healthcare services. These on-going interdisciplinary research efforts will significantly expand the applications of smartphone-based mHealthcare and telemedicine technologies and pave the way to the next generation of personalized healthcare monitoring and management. SBDAs will enable the creation of a learning environment, where the end-users analyse the *in vitro* diagnostic (IVD) results and adjust their lifestyle to achieve visible and sustained health benefits. Therefore, they will critically improve healthcare by empowering the patient to become an active decision maker.

The recent developments in mHealthcare and telemedicine have attracted considerable attention toward the use of smartphones as a platform for various mobile healthcare applications. There has been a significant increase in funding and a rapid growth in public interest for improved healthcare and better health management tools through various mHealthcare technologies. The most recent FDA clearance of the AliveCor Heart Monitor platform is a typical case of such significant achievements, which may inspire the development of other commercially-viable SBDAs in the coming years. Current efforts especially focus on overcoming the technical challenges associated with miniaturization, clinical validation, reproducibility, robustness, adaptability to various and rapidly evolving models of smartphones and compliance with industrial and healthcare requirements. The FDA-cleared LFA test strips are already commercially available for several disease-related biomarkers (such as cardiology, oncology, infections, hormone dysbalance and drug screening). Similarly, most of the POC glucose meters are also FDA-cleared. Therefore, the integration of such FDA-cleared or CE-marked devices with SBDA might enable the creation of new commercially-viable smartphone-based products.

The described commercial SBDAs are being used by several millions of smartphone users worldwide, which have led to critically improved and sustainable health outcomes by providing better healthcare monitoring and management tools. This will lead to significant cost-savings, as the users can participate in effective disease prevention and management, such as for diabetes, obesity, coronary heart disease and even cancer, by lifestyle adjustment and nutritional intervention/selection. Ideally, SBDAs may contribute to the diminution of the financial burden for such Western lifestyle-associated diseases. Healthcare can be revolutionized by providing gigantic data via centralized cloud computing, to establish enriched information for more predictive healthcare monitoring and management in societies.

The recent developments in cloud computing require a special mention here. Recent years have led to considerable advances in cloud computing for mHealthcare that can provide significant cost savings by reducing infrastructure costs [92]. However, this has given rise to critical concerns about the security and privacy of personal data [93,94]. Most nations have laws to physically store the data within the national boundaries. Therefore, significant research efforts are required in the coming years that will enable cloud computing to comply with these essential preliminary requirements [95]. Amazon Web Services now allows companies to store the data within national boundaries. The Government Cloud product from Google also counteracts this limitation by enabling governments to store data as per their national data security guidelines. Recent years have emphasized the need for the creation of international cloud computing standards. Several initiatives, such as EuroCloud and Google’s Data Liberation Front, have been started to establish such standards. Similarly, there are concerns over the privacy of personal information data in personal health records (PHRs), as these could be exposed to third party servers and unauthorized parties. The ethical guidelines clearly state that the patients should have full control over access to their own PHRs. Therefore, many strategies, such as attribute-based encryption [96] and employing a trusted third party [95], are being developed to address this concern. Several cloud computing models are being established to deal with the regulatory requirements of security and privacy [97]. The benefits of employing electronic health records (EHRs) in improving caregivers’ decisions and patients’ outcomes have been widely demonstrated [98]. The Health Information Technology for Economic and Clinical Health Act (HITECH) in the United States has further authorized incentive payments through insurance agencies to the healthcare practitioners who will use EHRs to achieve specified improvements in care delivery [99]. Smartphone-based mobile cloud computing [100], being employed in the developed SBDAs, will provide the desired next generation of mHealthcare technology for personalized healthcare needs [101].

It is highly expected that the next decade will witness a tremendous increase in the features of the smartphones, the next generation of mobile cloud computing and the contributing technologies. The much-awaited launch of iOS 8 in 2014 might include fitness and health tracking integration as a key feature in addition to the dedicated Healthbook application. Among various emerging breakthrough technologies, foldable screens and three-dimensional tracking will be integrated into the smartphone. Simultaneously, the cost of a smartphone continues to decrease owing to the growing number of smartphone users and Internet users. Such positive developments will pave the way toward improved healthcare in developing countries.
